# Adult tissue-resident stem cells—fact or fiction?

**DOI:** 10.1186/s13287-021-02142-x

**Published:** 2021-01-21

**Authors:** Deepa Bhartiya

**Affiliations:** Stem Cell Biology Department, ICMR-National Institute for Research in Reproductive Health, Jehangir Merwanji Street, Parel, Mumbai, 400012 India

**Keywords:** Adult stem cells, Single-cell RNAseq, Very small embryonic-like stem cells, Cancer stem cells, Dedifferentiation, Plasticity, Pluripotent

## Abstract

Life-long tissue homeostasis of adult tissues is supposedly maintained by the resident stem cells. These stem cells are quiescent in nature and rarely divide to self-renew and give rise to tissue-specific “progenitors” (lineage-restricted and tissue-committed) which divide rapidly and differentiate into tissue-specific cell types. However, it has proved difficult to isolate these quiescent stem cells as a physical entity. Recent single-cell RNAseq studies on several adult tissues including ovary, prostate, and cardiac tissues have not been able to detect stem cells. Thus, it has been postulated that adult cells dedifferentiate to stem-like state to ensure regeneration and can be defined as cells capable to replace lost cells through mitosis. This idea challenges basic paradigm of development biology regarding plasticity that a cell enters point of no return once it initiates differentiation. The underlying reason for this dilemma is that we are putting stem cells and somatic cells together while processing for various studies. Stem cells and adult mature cell types are distinct entities; stem cells are quiescent, small in size, and with minimal organelles whereas the mature cells are metabolically active and have multiple organelles lying in abundant cytoplasm. As a result, they do not pellet down together when centrifuged at 100–350*g*. At this speed, mature cells get collected but stem cells remain buoyant and can be pelleted by centrifuging at 1000*g*. Thus, inability to detect stem cells in recently published single-cell RNAseq studies is because the stem cells were unknowingly discarded while processing and were never subjected to RNAseq. This needs to be kept in mind before proposing to redefine adult stem cells.

## Main text

Current omics approaches require homogenization of cells to study their contents, and it is impossible to study stem cell dynamics by these approaches since the stem cells comprise less than 1% of total cells in adult tissues. Single-cell analysis to delineate cellular transcriptome at single cell level was declared as the breakthrough of the year 2018 by the journal *Science*. It was postulated that single-cell RNAseq could transform the basic biology and medical research landscape in the next 10 years. However, it seems this advance has led to considerable misperceptions in the field of stem cells rather than offering clarifications**.** Using single-cell analysis, recent reports in leading journals have denied presence of stem cells in adult human ovarian cortex [1], murine and human prostate [2], and murine cardiac tissue [3]. It has been suggested that mature cells in adult organs like the kidneys, lungs, liver, heart, pancreas, and prostate have the ability to dedifferentiate to stem-like state and participate in regeneration. Stem cell biologists have challenged basic concepts of cell plasticity, primarily because of their inability to isolate stem cells as a physical entity and now wish to redefine adult stem cells [4, 5]. Based on earlier reports, adult tissues are expected to harbor two populations of stem cells [6] including quiescent stem cells that undergo rare asymmetrical cell divisions to self-renew and give rise to slightly bigger-sized progenitors that become lineage-restricted, which divide rapidly by symmetrical cell divisions and clonal expansion before initiating differentiation into tissue-specific cell types. But Post and Clevers [7] recently proposed to redefine adult tissue stem cells based on their function to undergo mitosis to replace lost cells.

**Fig. 1:**
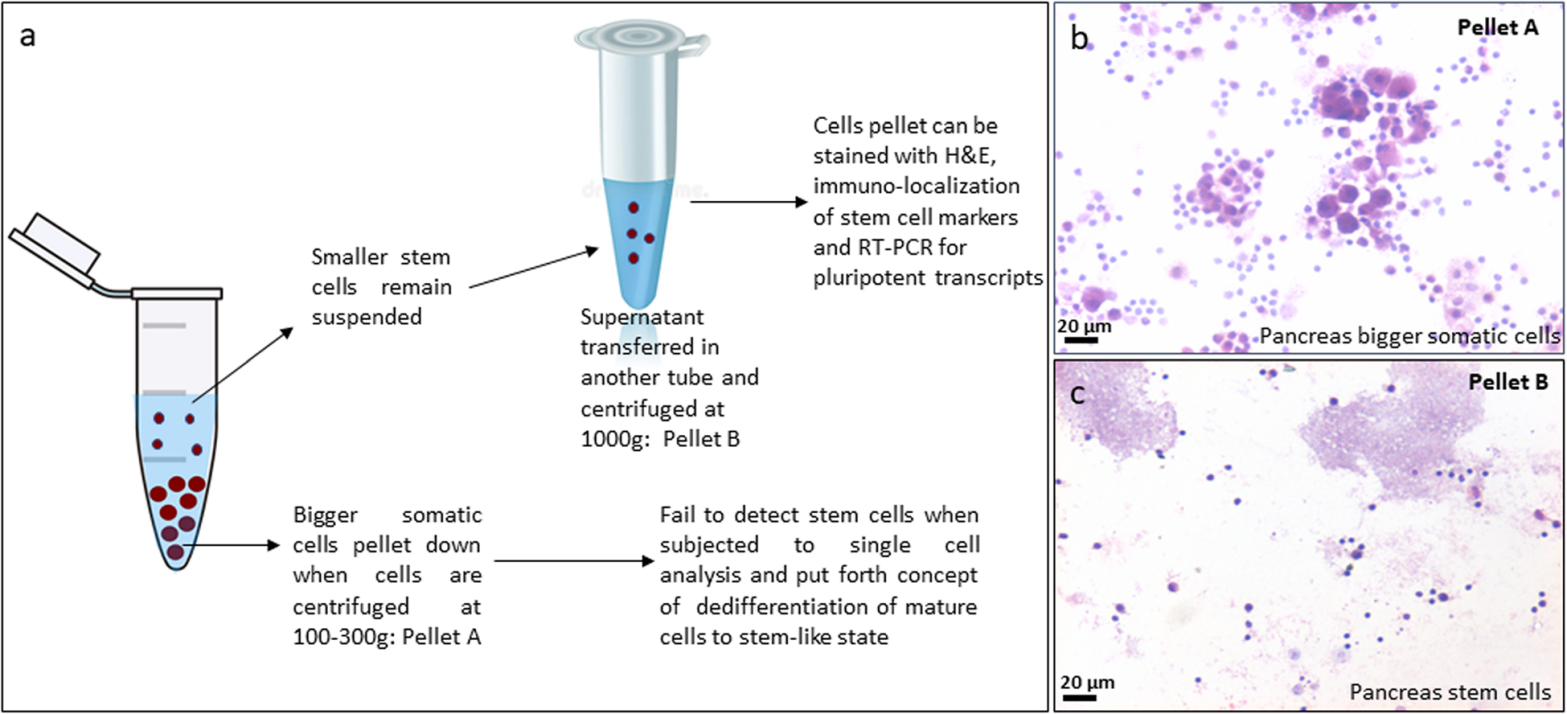
Stem cells in adult pancreas. **a** Stem cell enrichment from adult pancreas by centrifuging at higher speed. Adult mouse pancreas was subjected to enzymatic digestion for preparing a single cell suspension. **b** Upon centrifugation at 250*g*, large numbers of cells of different sizes with abundant cytoplasm and pale stained nuclei were observed in the cell smear after H&E staining. **c** On further centrifuging the supernatant at 1000*g*, putative, spherical stem cells with high nucleo-cytoplasmic ratio and darkly stained nuclei are clearly visualized. These stem cells can be further characterized by flow cytometry, immuno-localization and RT-PCR as published earlier [9]. **b** and **c** have been reproduced from our original article [9] with permission from Springer Nature

It becomes vital to appreciate the difference between stem cells and mature cells. Mature cells are bigger in size, are metabolically active, and have abundant cytoplasm holding large numbers of organelles to function normally, whereas stem cells are quiescent and much smaller in size with minimal cytoplasm and very few organelles. Stem cells remain buoyant when cell suspension (obtained after enzymatic digestion) from any tissue is spun at 100–350*g*. Somatic cells pellet down at this speed, and care is taken not to spin at higher speed; otherwise, they may get damaged and cytoplasm may burst leading to cell death. However, stem cells require higher speed of 1000*g* to pellet down [8–13] (Fig. 1). They are not damaged by higher centrifugation speed as they have minimal cytoplasm.

As shown in Table 1, various studies [1–3] used lower speed to pellet cells, and thus, stem cells were never subjected to RNAseq during single-cell analysis leading to negative findings. Similarly, Xiao et al. [14] centrifuged at 1200 rpm to process cells for multicolor flow cytometry experiments, discarded the stem cells unknowingly, and wrongly concluded that pancreas does not harbor stem cells. Similar technical mistake has been repeated in several published studies without realizing that they are inadvertently discarding stem cells.

**Table 1:** Centrifugation speed used to process cells for various studies

Tissue	Speed to process cells for studies	Reference
Ovary	Ovarian cell isolation at 300*g* for single-cell analysis	[1]
	Stem cells can be isolated by scraping ovary cell surface	[12]
Prostate	Prostate cell isolation at 200*g* for single-cell analysis	[2]
Cardiac	Cardiac cell isolation at 100*g* for single-cell analysis	[3]
Pancreas	Isolation of pancreatic cells and processing for multicolor flow cytometry at 1200 rpm	[14]
	Single cell suspension spun at 1000 rpm and then supernatant at 1000*g* helped collect VSELs and pancreas stem cells (PSCs) from both pancreas and the islets	[9]
Testes	Single cell suspension spun at 1000 rpm and then supernatant at 1000*g* to collect VSELs and spermatogonial stem cells (SSCs)	[11]
Uterus	Single cell suspension spun at 1000 rpm and then supernatant at 1000*g* to collect VSELs and endometrial stem cells (EnSCs)	[10]
Pancreas, spleen, lungs, gut epithelium, bone marrow, muscles	Single cell suspension spun at 1000 rpm and then supernatant at 1000*g* to collect stem cells	[8]

We had earlier reported that pluripotent very small embryonic-like stem cells (VSELs) in bone marrow/cord blood settle down with red blood cells upon density gradient centrifugation whereas stem cells are globally studied in the buffy coat [15]. Pluripotent VSELs in the red blood cells’ pellet were studied and characterized in-depth [16]. Ratajczak’s group from the University of Louisville, USA, reported VSELs initially in 2006, and now, > 30 independent groups have confirmed their presence [17]. These stem cells express pluripotent markers and markers specific for primordial germ cells (PGCs). Lineage and CD45− VSELs can differentiate into cells of all the 3 lineages, CD45+ HSCs, and also into germ cells [18]. Being quiescent (due to erasure of some paternally imprinted genes similar to post-migratory primordial germ cells), VSELs do not divide readily in vitro and survive both radiotherapy in mouse bone marrow [19] and chemotherapy in the testes [20, 21] and ovaries [22]. VSELs also survive in atrophied mouse uterus after bilateral ovariectomy [23]. Unlike hES/iPS cells that differentiate into their fetal counterparts, VSELs have the potential to regenerate adult tissues [24] and possibly also have a role to initiate cancers [25].

Essentially centrifuging cell suspension at 100-350*g* allows the majority of mature cells to pellet down, and later centrifugation of the supernatant at 1000*g* allows enrichment of stem cells. Using this simple and robust approach that can be easily replicated by any lab, VSELs and slightly bigger “progenitors” can be enriched in multiple adult tissues [8–13]. Appreciating and incorporating this crucial detail during processing to study stem cells will ensure their detection by single-cell analysis and will empower adult stem cell biology in normal and cancerous tissues and regenerative medicine to progress convincingly.

Similar to stem cells in adult tissues, there is still lack of clarity on the stem cells in cancer tissues (CSCs). The concept of CSCs was proposed 4 decades ago, but their identification and eradication have not been achieved as yet as was hoped initially. It was recently suggested by Clever’s group that CSCs also do not necessarily have to be rare and quiescent like the adult tissue-resident stem cells [26]. Logically, CSCs are the normal tissue stem cells that start functioning abnormally (uncontrolled proliferation) rather than their normal function of remaining quiescent and functioning in a subtle manner to maintain homeostasis. Have we missed out on CSCs on similar grounds and for similar reasons like the adult stem cells? Have CSCs been discarded unknowingly by centrifuging cells at lower speeds? This remains to be studied. Despite decades of research, we still do not understand how cancer initiates. Cancer incidence is increasing in current times, and new drugs are required for therapy that avoid recurrence. Tuveson and Clevers [27] discussed cancer modeling by use of organoids obtained by 3D culture as a good alternative for screening drugs for oncotherapy. But organoids are formed by actively dividing cancer cells and may not truly mirror the CSCs that are relatively quiescent in nature and survive oncotherapy. Our group recently showed that testicular cancer was initiated in adult mice, by treating them during neonatal life with diethylstilbestrol, due to excessive self-renewal of VSELs (increased seven folds) as confirmed by flow cytometry (cells were always centrifuged at 1000*g* to enumerate VSELs) along with their blocked differentiation due to disruption of NP95 expression [25]. This study provides first evidence that cancer results as a result of aberrant behavior of VSELs. This concept was earlier  proposed by Ratajczak’s group [28]. Ubiquitous expression of embryonic markers including OCT-4 and CD-133 on different types of cancers suggests that they all have a common origin from VSELs.

To conclude, wider acceptance and basic understanding of VSELs’ biology in normal and cancerous tissues is much required for future translation in the field of regenerative medicine and cancer biology.

## Data Availability

Not applicable

## Abbreviations

hES: Human embryonic stem cells
iPS: Induced pluripotent stem cells
VSELs: Very small embryonic-like stem cells
CSCs: Cancer stem cells
HSCs: Hematopoietic stem cells
